# Alcohol in the Workplace: A Qualitative Study of Employees' Experiences With Employer‐Supported Alcohol Treatment

**DOI:** 10.1111/dar.70194

**Published:** 2026-06-11

**Authors:** Isabella Pistone, Martina Martinez, Morten Sager, Kristina Berglund

**Affiliations:** ^1^ Department of Philosophy, Linguistics and Theory of Science University of Gothenburg Gothenburg Sweden; ^2^ Department of Psychology University of Gothenburg Gothenburg Sweden

**Keywords:** alcohol in the workplace, alcohol treatment, alcohol use disorder, employer‐supported alcohol treatment, qualitative research

## Abstract

**Introduction:**

Alcohol use disorder is a major public health issue, impacting health, well‐being, relationships and work performance. In Sweden, employers are legally required to provide access to and cover the cost of alcohol treatment for individuals with alcohol use disorder. Employer‐supported alcohol treatment is therefore rather common in Sweden; however, research on employees' experiences with this type of treatment remains unexplored. This qualitative study investigates how a group of Swedish employees who had received employer‐supported alcohol treatment experienced workplace management before, during and after treatment.

**Method:**

Interviews with 15 participants were analysed using thematic analysis, revealing three key themes.

**Results:**

The first theme highlights that many participants viewed their employer as a lifeline, experiencing relief when they received support to seek help for their alcohol problems. The second theme captures employees' reliance on their employer's ability to handle the situation, emphasising the crucial role managers play in the process, with varying levels of competence and support. Third, participants often felt alone upon returning to work, facing a discrepancy between their expectations of support and the actual workplace support provided post‐treatment.

**Discussion and Conclusions:**

The findings show how employer‐supported treatment may facilitate access to care and workplace reintegration under relatively favourable circumstances, particularly when supported by managerial competence, clear routines and continued follow‐up.

## 1 Introduction

Harmful alcohol use and alcohol use disorder (AUD) are widely recognised as significant public health issues that affect individuals' health, well‐being, social relationships, economic stability and work performance [1]. Excessive alcohol use is associated with serious health risks, including diseases such as cancer and liver cirrhosis, as well as an increased likelihood of injuries from incidents such as traffic accidents and violence. In response to these concerns, the United Nations and the World Health Organization have established strategies to address hazardous alcohol use. The World Health Organization's action plan for reducing harmful alcohol use (2022–2030) specifically identifies the workplace as a key setting for alcohol prevention [2].

The workplace is considered by many to be an ideal setting for alcohol use prevention [3, 4, 5]. The workplace setting allows a broad adult population to be reached, as most adults are employed and spend a substantial amount of time at work. This provides opportunities to identify problem drinking, offer preventive programs and promote healthy behaviour [4]. Employment plays a central role in the lives of many adults, providing income, social status and a sense of purpose. Alcohol misuse can jeopardise these aspects, resulting in serious consequences, including job loss. Employers, therefore, hold a unique influence and can potentially motivate employees to seek help for alcohol‐related problems [5].

A range of workplace alcohol prevention strategies spans a continuum from universal initiatives for the general employee population (primary prevention) to treatment, rehabilitation and return‐to‐work support for employees with AUD (often described as tertiary prevention/support). Unlike studies of universal workplace health promotion or screening interventions, this study concerns employer involvement after alcohol‐related problems had become sufficiently serious to warrant treatment and rehabilitation. The focus is therefore not on prevention in the narrow sense of reducing alcohol consumption among the general workforce, but on how the workplace manages treatment access, rehabilitation responsibility and return‐to‐work support for employees with established alcohol‐related problems. Recent research highlights the potential of workplaces to actively support employees' recovery from AUD [4]. Research on workplace programs for alcohol prevention suggests that these programs hold considerable promise. A recent meta‐analysis revealed that universal and selective interventions within the workplace significantly reduce employee alcohol consumption [6]. Similarly, a systematic review provided evidence supporting workplace‐based health promotion interventions, targeted brief interventions and universal substance use screening as effective strategies for reducing alcohol‐related problems [7]. While there is a growing body of research on universal and selective interventions, studies focused on interventions specifically targeting employees identified with AUD are still scarce.

In several countries, workplace responses to employee alcohol problems have historically been organised through employee assistance programs [8], which in many settings have developed from an initial focus on alcohol and substance use to a broader focus on mental health, family problems and general employee wellbeing [9]. One implication of this broader framing is that alcohol problems may receive less explicit attention as workplace and rehabilitation issues. By contrast, the Swedish labour market context gives employers a comparatively explicit role in rehabilitation when alcohol‐related problems affect work ability.

Swedish employers have a well‐established legal responsibility to initiate and support rehabilitation when AUD is identified among employees. This obligation, which has remained largely unchanged since 1982, is grounded in Swedish Labour Law, where AUD is recognised as a disease (ad 1982 nr 133) [10]. Employers are therefore required to investigate health‐related problems and implement reasonable measures to support rehabilitation and maintain work ability; consequently, individuals with a medical condition, such as AUD, may not be dismissed on the basis of their illness and must instead be offered support for rehabilitation. Moreover, there are no nationally standardised procedures specifying how workplaces should identify and manage alcohol‐related problems among employees. Instead, routines are typically developed at the organisational level and may vary considerably between workplaces; larger organisations often have more formalised policies, whereas smaller workplaces may rely on less standardised practices. Similarly, there is no nationally standardised program requiring managers to be trained in how to handle employee alcohol‐related problems; training and guidance may be offered locally (e.g., via occupational health services or external providers), but their availability and uptake vary across workplaces.

In practice, an employee is typically considered in need of treatment once harmful use and/or AUD has been identified through health care services. When a treatment need is identified, the employer's statutory responsibility is to initiate treatment. At the same time, this comparatively explicit rehabilitation role should not be interpreted as guaranteeing successful long‐term recovery. AUD is often a chronic or recurrent condition, and recovery may involve relapse, continued support needs and varying degrees of work ability over time. Thus, even in a context where employers may facilitate access to treatment and rehabilitation, such support should be understood as one possible route to support rather than as an assured solution to alcohol‐related problems.

Similarly, employers' rehabilitation responsibilities should not be understood as unlimited tolerance of ongoing alcohol‐related misconduct or unsafe behaviour. In practice, employers must balance rehabilitation obligations with work ability, safety and the requirements of the employment contract. Continued drinking, repeated relapse, refusal to participate in reasonable rehabilitation measures, or behaviour that creates risks for the employee, colleagues, clients or the public may lead to further managerial and legal measures. The present study does not examine such cases, and therefore cannot address how employers manage situations where alcohol problems continue despite intervention.

In this paper, we use the term employer‐supported treatment to refer to workplace‐facilitated access to alcohol treatment and rehabilitation (e.g., initiating contact, referral, coordination with services, workplace accommodations and follow‐up). In Sweden, a common workplace response for employees with severe alcohol‐related problems is referral to a treatment provider. Employers are not legally required to finance treatment; employees may also access standard treatment options through primary health care (including referral to specialised treatment) and psychosocial treatment through social services, and private treatment facilities are available (funded by social services in some cases or paid for privately by the individual). In the present study, all participants received treatment that was financed by their employer. While employers are not legally obligated to cover treatment costs, it is common for them to assume the expense, either as a deliberate support measure or due to a mistaken belief that such an obligation exists [11]. Despite the increasing relevance of employer‐supported alcohol treatment, research remains limited, particularly regarding how employees experience this form of employer intervention.

To address this gap, we conducted an exploratory qualitative study to gain insights into how employees experience management in the workplace when receiving employer‐supported alcohol treatment. Qualitative methods enable a closer examination of employees' personal accounts, providing a nuanced understanding of their perspectives on employer interventions. In this study, we thus take an open approach to explore the experiences of employees with AUD who have received employer‐supported treatment, with a focus on the management of their situation by their employer and the personal journey they undergo from the disclosure of their alcohol problem through the treatment process and eventually returning to work after treatment.

To guide our analysis, we formulated the following broad research question: *How do employees with AUD experience receiving help for treatment from their employer?* This study thus aims to contribute insights into the role of employer‐supported treatment in facilitating employees' recovery from AUD as an employer‐led intervention for alcohol problems in the workplace. It is important to note that the present study does not aim to represent the experiences of all employees with AUD in Sweden. Rather, it explores accounts from employees who received employer‐supported treatment, remained connected to employment and were able to reflect retrospectively on this process in an interview. The study therefore provides insight into employer‐supported treatment under relatively favourable circumstances, while identifying elements of support, management and follow‐up that participants experienced as important for treatment access and workplace reintegration.

## 2 Methods

This study is part of a larger research project that aimed to examine alcohol in the workplace through a mixed‐method approach with an explanatory sequential design. This larger project was initiated in 2020 and aimed to investigate employees' perceptions of receiving treatment via their employer, as well as work‐related factors that are associated with alcohol use and alcohol prevention. Unfortunately, due to the COVID‐19 pandemic, the quantitative part of the project had to be cancelled. However, the researcher decided to revise the project and carry out qualitative interviews; the interviews on which this study is based. The ethical approval for this study was given during year 2018 (Exp. 2018‐12‐11, 1016‐18) by the ethics committee the ‘Central Ethical Review Board’ in Gothenburg. All participants in this study gave informed consent regarding participation. In this study, we employed a qualitative thematic analysis methodology [12]. Through its theoretical freedom, thematic analysis provides a flexible and useful research tool that can provide a rich, yet detailed and complex, account of the research topic. It is a particularly useful method when investigating an under‐researched area, as it enables a rich thematic description of the phenomenon and helps identify predominant or important themes [12]. An inductive approach was applied throughout the study, with the interview guide refined during data collection and themes identified through inductive coding. This meant that the development of themes was strongly linked to the data instead of fitting the data into a preexisting coding frame or theoretical framework.

### 2.1 Data Collection and Participants

In depth, semi‐structured, audio‐recorded interviews were conducted with 15 participants. The interviews allowed exploration of participants' experiences through a flexible dialogue in which questions could be adapted and topics pursued as they emerged [13]. The interviews followed a loosely structured guide covering participants' experiences of being referred to treatment for AUD, experiences of how the employer handled the situation and how they perceived being reintegrated in the workplace after treatment. In this study, all participants' treatment was financed by their employer. The analytic focus was on employees' experiences of employer involvement and workplace reintegration surrounding treatment. All the interviews were transcribed verbatim. The participants were assigned pseudonyms to ensure anonymity. The interviewer had no prior relationship with the participants.

All participants had undergone treatment for AUDs at the same occupational alcohol and drug treatment provider in western Sweden. The provider is a non‐profit foundation established in 1987 by several large employers in the region to offer assessment, treatment and rehabilitation services for employees, operating on a not‐for‐profit basis. The participants were recruited through two routes. First, we contacted individuals who had responded to a survey within the larger research project and had reported interest in being interviewed; this resulted in the recruitment of 10 participants. Second, the treatment provider identified eligible patients in its database and distributed study information to individuals who had completed treatment (including aftercare) within the past 2 years, as well as those in the final phase of aftercare and nearing completion of outpatient treatment. Five additional participants were recruited through this route. We aimed to include participants from diverse life situations to ensure a breadth of perspectives and a nuanced understanding of the research topic. However, recruitment relied on voluntary participation and self‐referral: invited individuals received written information and contacted the research team if they wished to participate. As participation required willingness and sufficient stability to take part in an in‐depth interview, the sample may primarily reflect the experiences of those who were able and willing to participate; this is further addressed in the Discussion.

### 2.2 Analysis

We conducted an inductive thematic analysis following Braun and Clarke's six‐phase approach [12]. The transcripts were read repeatedly for familiarisation. The data were coded inductively, and the codes were collated into candidate themes, which were reviewed and refined through iterative discussions within the research team. MaxQDA was used to support data management and coding. The final themes were defined, named and illustrated with representative quotations.

## 3 Results

The participants' ages ranged from 34 to 71 years, with a mean age of 54 years. Nine patients were male, and six were female. The years since they underwent treatment vary from less than a year ago up to 20 years ago. Some of the participants did not think that their employer had noticed the alcohol problem before it was revealed. Others were convinced that their alcohol problems were already known before it was disclosed. For all the participants, the alcohol problems had been ongoing for some time and then escalated, leading to either self‐referral or confrontation by the immediate manager. While most participants did not view their work situation as a cause of their alcohol problems, some reported that aspects of the workplace, such as dysfunction, unclear roles or an unhealthy drinking culture, may have played a contributing role. In the following section, we elaborate on the three main themes identified in the thematic analysis. Table 1 provides an overview of the themes and subthemes.

**TABLE 1:** Overview of themes and subthemes.

Themes	Subthemes
The employer as a lifeline: disclosing the alcohol problem and navigating relief, uncertainty and shame	1.1 The employer as a lifeline to recovery
	1.2 An emotional whirlwind after disclosure of the alcohol problem
2 Dependence on the employers' ability to handle the situation	2.1 The management played a significant role
	2.2 Knowledge about how to handle alcohol problems
3 Alone when returning to the workplace: discrepancy between expectations	—

### 3.1 Theme 1: The Employer as a Lifeline: Disclosing the Alcohol Problem and Navigating Relief, Uncertainty and Shame

A prominent theme identified in the analysis was the crucial role employers played in enabling participants to receive treatment for alcohol problems. For many, the employer was not just a pathway to help but often the only viable option. A strong relationship with their manager was pivotal, fostering trust that made it possible for participants to disclose their alcohol problem. Seeking help from the employer often brought a sense of relief, as it meant that the participants no longer had to bear the burden alone. However, this event was also accompanied by feelings of shame, vulnerability and uncertainty about how their employment might be affected.

#### 3.1.1 Subtheme 1.1: The Employer as a Lifeline to Recovery

For many participants, their employer represented the first, and often the only, source of support for managing their alcohol problems. Without the financial means to afford treatment independently and limited awareness of other options, employer‐supported treatment was seen as indispensable. The participants frequently disclosed their alcohol problems to their manager, often encouraged by family members, who pressured them to address their drinking. One participant explained:

I mean, I don't think there was any other option for me than to go and talk to my employer. It was mostly because my partner wanted me to ask for help. Without the help I got from my employer, I wouldn't be sober today. (Andreas, 55, healthcare worker)

The participants often described their manager as a lifeline, providing essential support when they felt desperate and unsure of how to seek help. Some participants initiated a meeting with their manager to disclose their alcohol problems, whereas others waited for the right moment, choosing to open up when their manager asked how they were doing. For these participants, the manager's inquiry created a natural opportunity to share their problem. In some cases, managers took the initiative to confront participants about their alcohol problems. The participants who experienced this intervention expressed deep gratitude, often describing it as lifesaving. Reflecting on such an instance, one participant shared:

And in the end, my manager had seen the signs. I had a good relationship with him for many years. We talked a lot. Eventually, he just called me in and simply asked, ‘How are you truly doing? Are you drinking too much? Using drugs?’ And I admitted it. I said, ‘Yes, I am. I'm drinking myself to death’. (Albin, 57, actor)

This account highlights the importance of trust in the manager–employee relationship, which enables participants to openly admit their problems and accept help. Across the interviews, the quality of the relationship with the manager emerged as a critical factor. Whether participants approached their manager or were confronted, a supportive and understanding manager played a vital role in encouraging openness and facilitating access to treatment.

#### 3.1.2 Subtheme 1.2: An Emotional Whirlwind After Disclosure of the Alcohol Problem

Many participants described experiencing a profound sense of relief once their alcohol problem became known, whether they themselves sought help from their employer or were confronted by their manager. As one participant explained,

Throughout this whole change, I admitted that I couldn't handle it. I just had to let others take over, so it was like … I couldn't help myself, and as for what I felt during all of it, I don't truly know. (Britta, 44, healthcare worker)

The relief expressed by the participant stemmed from recognising that she no longer had to face the struggle alone and echoes a common experience expressed by the participants. The involvement of their employer allowed participants to feel supported, as someone else took charge of handling their alcohol problem.

However, not all participants felt immediately positive about the employer's actions to provide treatment. Some described initial feelings of anger and frustration, particularly when they perceived the employer as ‘forcing’ them into treatment. These participants often believed that their alcohol problem was exaggerated or that they felt misunderstood and had expected other forms of less comprehensive support from the employer. Over time, however, this anger often evolved into gratitude, as participants came to appreciate their employer's recognition of the alcohol problem and decisive action.

Most participants expressed gratitude towards their employer, but their narratives also revealed strong feelings of shame and vulnerability. This was especially pronounced in the period immediately after their alcohol problems became known and before treatment began. One participant shared:

I couldn't protest against it, because it was so incredibly shameful, and I felt like a wet spot, just listening to what they said. It was probably the worst thing that had ever happened to me in my life. Because fundamentally, I am a very functional and capable person. However, I was very sick. (André, 40, healthcare worker)

Many participants echoed these sentiments, describing themselves as feeling small, helpless and exposed. Acknowledging their dependency on their employer often intensifies these emotions, creating a mix of relief and shame. While most participants wanted help, they felt powerless to negotiate or demand anything beyond what the employer offered. Additionally, some participants expressed uncertainty about the potential consequences of disclosing their alcohol problems at work. They were unsure about the legal or professional implications and feared jeopardising their employment. This complex interplay of emotions, relief, anger, vulnerability, shame and uncertainty, underscores the nuanced and often challenging dynamics between employees seeking help and employers offering treatment.

### 3.2 Theme 2: Dependence on Employers' Ability to Handle the Situation

The participants' experiences with the process, from the disclosure of their alcohol problems to receiving treatment, varied widely. While many described their employer's ability to handle the situation in positive terms and expressed satisfaction with the process, others faced challenges, such as the ability to recall a prolonged and demanding process to access treatment. In this context, we describe the important role that employers play in this process beyond offering financial support for treatment and how knowledge about alcohol problems is crucial for being able to provide the right support.

#### 3.2.1 Subtheme 2.1: Management Played a Significant Role

A recurring pattern in participants' accounts is how important the management around the referring to treatment was. The role of the immediate manager was particularly pivotal, shaping participants' experiences during this period. As noted in the previous theme, disclosing an alcohol problem often left participants feeling exposed, ashamed and insignificant. The way the employer managed the process from disclosure to treatment influenced whether the participants viewed the experience as supportive or distressing.

Positive experiences are characterised by empathetic and proactive management, which creates a safe and supportive environment, facilitating a smoother path towards treatment. One participant explained that an important factor contributing to her sense of safety and support was her manager's ability to separate her from her alcohol problem. She emphasised how critical it was that the manager did not judge her on the basis of her addiction but instead made a clear distinction between her as a person and her illness. This distinction was especially important to her, as she felt deep shame about her alcohol abuse and was concerned that disclosing her problem might result in her not being taken seriously at work. Her manager's approach made it easier for her during this challenging period before she entered treatment. Another participant, who also experienced great support from her manager, described how the manager dedramatised her AUD while simultaneously taking it seriously and taking prompt action:

She never hesitated when I told her; it was immediately 'We're going to solve this'. There was none of that 'Oh my God, do you have a problem?' Instead, it was, 'No worries, we'll figure this out. If you're on board, we'll work together'. It was the same with everything else—'Just tell me what you need, and we'll try to sort it out'. She would even come out to the department and say, 'Hi, how are you doing today?' (Barbro, 52, healthcare worker)

The participant felt treated with empathy, understanding and a sense of being taken seriously after disclosing her alcohol abuse to her manager, which is a recurrent pattern in many of the participants' accounts. In their reflections on this period, participants highlighted these factors as playing a significant role in alleviating the challenges of an otherwise unsettling time. Another factor identified as important was the manager's firm approach, as well as clear and consistent boundaries with the participant:

My employer was very strict with me, and I believe that was one of the reasons I managed to get through this. I do not think I would have made it if they hadn't been so firm with me. (Beata, 71, manager)

The quote highlights two key aspects: clear and firm management as well as the importance of the employer's role in participants' recovery. This participant underscores that he did not believe that he would have managed to become sober without the help and strict guidance of his employer.

Conversely, negative experiences arise when the employer lacks the will, sensitivity or ability to provide adequate support, causing participants to feel frustrated and misunderstood. Those participants who did not have a positive experience during the period before entering treatment often faced long and chaotic processes, with many years passing from the disclosure of their AUD to being referred for treatment. They described this as a prolonged and difficult process, with several years elapsing between the employer's initial awareness of the alcohol problem and the suggestion of treatment. One participant waited 10 years after he first discussed his AUD with his manager. He described these years as chaotic, marked by numerous incidents that he experienced as deeply offensive:

We had a workplace meeting, and my colleagues had been informed that there was going to be a discussion about me. However, I didn't know about it. In addition, they didn't know what it was going to be about either, as I determined afterward. Strangely enough, I already sensed that something was going on when I arrived at the meeting. In any case, my manager then told my five colleagues that I have a problem with alcohol and that I was going to be reassigned. That came as a complete shock to me as well. I hadn't been involved in this information and didn't know I was going to be reassigned. Some of my colleagues started crying. I did too. I was so upset. It was a harsh and sad meeting. (Anders, 58, social care worker)

The participant experienced this situation as deeply disrespectful and described feeling diminished, exposed and saddened by the way his manager treated him during this time. Despite his openness about his AUD, he perceived his manager as being more interested in removing him than in offering support.

As illustrated in the quote above, several participants felt that they were not being listened to and were excluded from decisions about their alcohol recovery. Many linked this experience to already having a poor relationship with their immediate manager, which, they felt, led the manager to be less inclined to offer support. These participants described their managers as failing to provide support, being unsympathetic and inconsiderate:

I was told before I went there that I should realize how expensive this was. That I was costing so much money. After the treatment was finished, I even received a call from a cheerful operations manager saying that most of it was deductible. I ended up not being so expensive. (Anders, 58, social care worker)

As shown in this quote, the participants felt reduced to a problem for their employer, and the way their managers handled the situation made them feel belittled and overlooked during an already vulnerable time.

#### 3.2.2 Subtheme 2.2: Knowledge About How to Handle Alcohol Problems

Knowledge of alcohol‐related problems and how employers can manage them was a recurrent theme in the participants' narratives. Most participants, regardless of whether they had a positive or negative experience with how their manager handled the period before they entered treatment, felt that their employer lacked knowledge about how to address alcohol problems in the workplace. Participants who had a good relationship with their manager and felt met with empathy reported that, although their manager lacked knowledge about how to handle the situation, they nevertheless tried to manage it to the best of their ability. However, many participants thought their manager could have handled it better if they had more knowledge and clearer routines:

I believe it's a lack of knowledge, and I can understand that because when you're working with people who don't have any alcohol addiction or substance abuse issues, you don't know how it works. You don't know what to do. So, I don't want to blame them either. (André, 40, healthcare worker)

The participant acknowledged that his employer lacked knowledge about alcohol addiction and was consequently unable to manage the situation as he would have preferred. At the same time, he recognised that it may be unrealistic to expect managers, who typically do not deal with AUD, to have expertise in this area and therefore he did not blame them. This echoes many other participants' accounts, where they excuse their manager's lack of knowledge by explaining that it might be too much to demand that they have knowledge about alcohol problems.

One participant reported that his employer had good knowledge of how to handle employees with alcohol problems*:*

[The employer] has good routines once you actually want it, then the routines kick in truly well. We have a fantastic occupational health service with people who are very knowledgeable and very attentive in every possible way. And if you choose openness like I did, then you get support from all your colleagues in every situation, so for me, it's been, I wouldn't say a smooth journey, but it has been fairly easy. (Assar, 55, manager)

This participant thus experienced that a combination of good routines and knowledgeable staff made a difference, facilitating his recovery process.

### 3.3 Theme 3: Alone When Returning to the Workplace: Discrepancy Between Expectations

The treatment offered at the centre where all participants were treated for their alcohol problems included either a 1‐month residential program or a 1‐year outpatient program with weekly meetings. After completing treatment, several participants expressed feeling unsupported by their employers. They perceived that their employers considered the issue ‘resolved’ once treatment was completed, whereas the participants themselves felt that the real challenge had ‘had just begun’:

Now you're healthy. Now you're cured. No, you're never cured of alcoholism or drug addiction. Once an addict, always an addict […] you have to keep following up all the time. They were like, ‘Well, now you must be cured. Now you can start working normally again.’ No, it's not that simple. (Albin, 57, actor)

This sentiment was common among participants. For them, treatment marked the start of a completely new way of living, and reintegration into the workplace was often challenging. Many participants felt isolated in managing their new reality and believed that they needed more support than they received:

The worst time was actually after [treatment] when I was back, because everyone around me thought it would be fine now […] I felt very alone in dealing with this. (André, 40, healthcare worker)

The participants mentioned various forms of support they felt were lacking during their reintegration into the workplace. While some wished for a clear follow‐up plan with their immediate manager after returning to work, others hoped for more simple gestures of support, such as their managers or colleagues asking how they were doing. The participants interpreted the lack of engagement from their employer and colleagues as a reflection of insufficient knowledge about how AUD works and a lack of understanding of how life‐changing sobriety was for them. Many employers appeared to view their primary responsibility as referring employees to treatment, believing that their role ended once treatment began. The participants, however, expressed that it was strange and disheartening that their recovery from AUD was not followed by the workplace. This highlights a discrepancy in expectations between employers and employees.

## 4 Discussion and Conclusions

To our knowledge, this qualitative study is the first to explore employees' experiences of how their AUD was managed in the workplace before and after employer‐supported treatment. By examining these experiences, this study provides valuable insights into alcohol prevention and rehabilitation in the workplace.

We identified three broad themes: (i) The employer as a lifeline: disclosing the alcohol problem and navigating relief, uncertainty and shame reflects the complex emotions employees experienced when revealing their alcohol problem at work. While some felt relief and support, others struggled with feelings of vulnerability and fear of stigma. (ii) Dependence on employers' ability to handle the situation captures the variability in how employers manage employees' alcohol problems, highlighting both effective support and cases where inadequate responses exacerbate employees' challenges. (iii) When returning to the workplace, the discrepancy between expectations reflects the challenges employees face upon reintegration, where expectations of a supportive and understanding work environment are often unmet, leading to feelings of isolation and uncertainty.

Overall, the findings show that employers can play a significant role before, during and after treatment, particularly in shaping employees' experiences of disclosure and reintegration. Importantly, our results highlight a mismatch in expectations regarding posttreatment support: participants often expected ongoing follow‐up and practical support when returning to work, whereas employers were described as viewing their role as largely completed once treatment had ended.

As Roman and Blum [5] argue, employment plays a central role in the lives of many adults, giving employers a unique position to support employees both in seeking help and throughout the rehabilitation process. The findings from this study further illustrate that how an employer managed the process, from disclosure to treatment, strongly influenced whether participants perceived the experience as supportive or distressing. The results of a study by Martinez et al. [14] indicate that managers valued gaining knowledge about harmful and hazardous alcohol consumption, as well as acquiring practical tools to approach employees with potential AUD. In Martinez et al.'s [14] study, the managers described a notable shift in how they understood the employer's responsibilities, indicating that they had not fully recognised their crucial role in alcohol prevention prior to the training. This aligns with the findings of the present study, which highlight that managers, according to the participants, often lacked the necessary knowledge to handle these situations effectively and provide a supportive work environment before and after employer‐supported alcohol treatment.

This discrepancy carries significant implications for workplace reintegration. Previous research has emphasised the role of managers' leadership styles in maintaining a safe workplace [15, 16] and promoting employee health [17, 18]. Managers have also been highlighted as key actors in facilitating alcohol problem prevention [14], and the significance of work in recovery has been described in qualitative research on substance use disorders [19]. In the present study, participants described limited follow‐up or understanding at work during the transition to sobriety and return‐to‐work. Several participants suggested that simple actions, such as regular check‐ins from managers or colleagues, could have made a substantial difference. Taken together, these findings underscore the importance of aligning expectations between employees and managers, as well as strengthening managerial competence in addressing alcohol‐related problems and supporting return to work practices.

Employer practices for handling alcohol‐related problems are likely to vary across workplaces, as policies, routines and managerial mandates are typically developed at the organisational level rather than being nationally standardised. Such contextual variation may help explain differences in how participants described their situations being handled. Notably, recent research suggests that clear managerial mandates to address alcohol‐related issues can facilitate workplace alcohol prevention, and that training and competence development in alcohol prevention are important for translating such efforts into practice [20].

A limitation of this study is that the sample was skewed towards participants in the later stages of their working lives, with several approaching retirement age. This may have shaped the accounts captured here, as long work experience and greater job security could influence both the willingness to disclose alcohol‐related problems at work as well as the perceptions of employer support. Future studies would benefit from including participants across early‐, middle‐ and late‐career stages to provide a more nuanced understanding of employer‐supported treatment and reintegration into the workplace. Taken together, these sample characteristics should be considered when the findings are interpreted.

Additionally, a limitation is potential self‐selection bias. Recruitment relied on voluntary participation, and taking part required willingness and sufficient stability to engage in an in‐depth interview. The sample may therefore overrepresent employees with more established recovery processes, more stable employment situations, and/or relatively constructive relationships with employers. Consequently, the perspectives of individuals with ongoing severe alcohol‐related problems, relapse, unstable living conditions, poorer outcomes (e.g., job loss or prolonged absence) or particularly adverse employer experiences may be underrepresented. The aim of this study was not to evaluate treatment effectiveness or long‐term outcomes but to explore employees' experiences with employer‐supported treatment and reintegration. The findings should therefore be interpreted as illustrating experiences among those who were able and willing to participate, rather than representing the full spectrum of employees with AUD. Consequently, the study cannot determine how common employer‐supported treatment is, how often it succeeds or what happens in cases where employees continue to drink, relapse repeatedly, lose employment or decline rehabilitation. Future research could examine the extent to which these patterns hold in broader samples, potentially via quantitative designs and include perspectives from managers who support employees with alcohol‐related problems.

Although the legal framework for employer responsibility for rehabilitation has remained largely stable in Sweden, participants' experiences span different time periods. Secular changes in workplace policies and practices, treatment pathways and social norms related to alcohol problems may therefore have influenced how experiences were managed and recalled; the findings should be interpreted as capturing cross‐temporal perspectives within this Swedish context rather than reflecting a single historical moment. Moreover, the temporal breadth of the material may also be considered a strength, as it allows us to capture experiences across different periods and workplace contexts. The recurrence of similar issues across accounts suggests that some challenges and support needs may persist over time within this setting.

## 5 Conclusion

In conclusion, this study highlights the workplace as an important setting not only for alcohol prevention in a broad sense, but also for employer‐supported treatment when employees have established alcohol‐related problems. By focusing on employees who received employer‐supported treatment, the findings show how workplace policies and managerial practices are experienced in practice, particularly during disclosure, referral to treatment and reintegration after treatment. The results suggest that employer‐supported treatment is experienced as meaningful when it is accompanied by trust, clear routines, managerial competence and continued follow‐up. At the same time, the findings point to the need for clearer expectations and structured support after treatment, as employees may experience a gap between formal access to treatment and everyday workplace reintegration. Rather than evaluating the effectiveness of workplace alcohol interventions in general, this study contributes valuable insight into how employment‐supported alcohol treatment is experienced by employees in practice.

## Author Contributions

Each author certifies that their contribution to this work meets the standards of the International Committee of Medical Journal Editors.

## Funding

This study was supported by the Research Council for ‘Systembolaget’ (The State Monopoly of Alcoholic Beverages) under Grant Exp. 2018‐12‐11 (1016‐18).

## Conflicts of Interest

The authors declare no conflicts of interest.

## Data Availability

The data that support the findings of this study are available from the corresponding author upon reasonable request.
